# Draft Genome Sequence of an NDM-5-Producing *Klebsiella pneumoniae* Sequence Type 14 Strain of Serotype K2

**DOI:** 10.1128/genomeA.01610-15

**Published:** 2016-03-17

**Authors:** Pan-pan Liu, Yang Liu, Lian-hui Wang, Dan-dan Wei, La-Gen Wan

**Affiliations:** Department of Bacteriology, First Affiliated Hospital of Nanchang University, Nanchang University, Nanchang, PR China

## Abstract

We report here the draft genome sequence of uropathogenic *Klebsiella pneumoniae* sequence type 14 strain of serotype K2 possessing *bla*_NDM-5_, isolated from a 65-year-old male in China without a history of travel abroad.

## GENOME ANNOUNCEMENT

*Klebsiella pneumoniae* is a major human pathogen causing hospital- and community-acquired infections (1, 2). The spread of NDM producers is now considered an endemic threat in *K. pneumoniae* and represents an important source of multidrug resistance around the world. *K. pneumoniae* sequence type 14 (ST14) has previously been described as a host lineage for the NDM-1 enzyme (3). Further, Serotype K2 has been described in one previous publication as frequently linked to ST14 (4).

Here, we report the unscaffolded whole-genome shotgun sequence of *K. pneumoniae* strain NUHL24835 recovered from a urine sample from a 65-year-old female patient hospitalized in the teaching hospital of Nanchang University, China, in 2015. The strain belongs to ST14 with a K2 capsular serotype and shows multiple resistances to clinically used antibiotics, including all β-lactams (ertapenem, meropenem, and imipenem showed MICs of >32 µg/ml), fluoroquinolones, aminoglycosides (except for amikacin), sulfonamides, and macrolides. It was susceptible to colistin and tigecycline.

The genomic DNA from *K. pneumoniae* NUHL24835 was sequenced by next-generation sequencing using an Illumina HiSeq 2000 instrument with a 2 × 151-bp paired-end approach. The draft genome of *K. pneumoniae* NUHL24835 comprises 5,387,996 bp, with a G+C content of approximately 58.53%. The protein-coding regions were predicted by Glimmer version 3.02 (http://www.cbcb.umd.edu/software/glimmer). In total, 5,191coding genes were identified, for a total length of 4,804,392 bp and 86.56% coverage of the genome. Eighty-four tRNA genes and 24 rRNA genes have putative functions assigned on the basis of the annotation.

The contigs were initially annotated using RAST (http://rast.nmpdr.org). A BLAST analysis and manual annotation utilized previously reannotated reference sequences and IS Finder (https://www-is.biotoul.fr). The MLST, ResFinder, and PlasmidFinder (http://www.genomicepidemiology.org) databases were used to characterize sequence typing, antibiotic resistance mechanisms, and the plasmid Inc types, respectively, of *K. pneumoniae* NUHL24835. ST14, plasmid Inc types of IncFII and IncX3, and the genes *bla*_NDM-5_, *bla*_CTX-M-15_, *bla*_TEM-1_, *qnrS1*, *aadA5*, *sul1*, and *dfrA17* were identified. This finding is consistent with previously reported experimental results (5). A comparative analysis of the genome from our isolate with published *K. pneumoniae* genomes will be reported in the future.

A screening for (putative) virulence genes present in the BIGSdb-Kp database (http://bigsdb.web.pasteur.fr/klebsiella/klebsiella.html), performed using the BLASTn tool, revealed (i) the *kfuABC* system (6), which is responsible for ferric iron uptake, and (ii) the mannose-resistant *Klebsiella*-like (type III) fimbriae cluster *mrkABCDFHIJ* (7). Interestingly, the allantoin operon was not present in the genome of the *K. quasipneumoniae* subsp. *quasipneumoniae* type strain, suggesting recent horizontal acquisition by *K. pneumoniae* NUHL24835. The strain possessed a capsular *wzi-*2/K2 allele. Notably, the *rmpA* gene, previously associated with the hypermucoviscous phenotype in *K. pneumoniae* strains (8), was found in the genome of *K. pneumoniae* NUHL24835, suggesting the presence of a different capsular regulation mechanism. The identified virulence determinants may have contributed to the infection and/or colonization of *K. pneumoniae* NUHL24835 in the urinary tract.

### Nucleotide sequence accession number.

The whole-genome shotgun project of *K. pneumoniae* NUHL24835 has been deposited at GenBank under the accession number CP014004.

## References

[1] Podschun R, Ullmann U 1998 *Klebsiella* spp. as nosocomial pathogens: epidemiology, taxonomy, typing methods, and pathogenicity factors. Clin Microbiol Rev 11:589–603. 9767057 10.1128/cmr.11.4.589 PMC88898

[2] Broberg CA, Palacios M, Miller VL 2014 *Klebsiella*: a long way to go towards understanding this enigmatic jet-setter. F1000Prime Rep 6:64. doi:10.12703/P6-64. 25165563 PMC4126530

[3] Woodford N, Turton JF, Livermore DM 2011 Multiresistant gram-negative bacteria: the role of high-risk clones in the dissemination of antibiotic resistance. FEMS Microbiol Rev 35:736–755. doi:10.1111/j.1574-6976.2011.00268.x. 21303394

[4] Brisse S, Fevre C, Passet V, Issenhuth-Jeanjean S, Tournebize R, Diancourt L, Grimont P 2009 Virulent clones of *Klebsiella pneumoniae*: identification and evolutionary scenario based on genomic and phenotypic characterization. PLoS One 4:e4982. doi:10.1371/journal.pone.0004982. 19319196 PMC2656620

[5] De Man TJ, Perry KA, Avillan JJ, Rasheed JK, Limbago BM 2015 Draft genome sequence of a New Delhi metallo-β-Lactamase-5 (NDM-5)-producing multidrug-resistant *Escherichia coli* Isolate. Genome Announc 3(2):e00017-15. doi:10.1128/genomeA.00017-15. 25744985 PMC4358372

[6] Ma L-C, Fang C-T, Lee C-Z, Shun C-T, Wang J-T 2005 Genomic heterogeneity in *Klebsiella pneumoniae* strains is associated with primary pyogenic liver abscess and metastatic infection. J Infect Dis 192:117–128. doi:10.1086/430619. 15942901

[7] Gerlach GF, Clegg S, Allen BL 1989 Identification and characterization of the genes encoding the type 3 and type 1 fimbrial adhesins of *Klebsiella pneumoniae*. J Bacteriol 171:1262–1270. 2563996 10.1128/jb.171.3.1262-1270.1989 PMC209739

[8] Hsu CR, Lin TL, Chen YC, Chou HC, Wang JT 2011 The role of *Klebsiella pneumoniae rmpA* in capsular polysaccharide synthesis and virulence revisited. Microbiology 157:3446–3457. doi:10.1099/mic.0.050336-0. 21964731

